# Neurodegeneration With Brain Iron Accumulation in a Case of Adult Aceruloplasminemia

**DOI:** 10.7759/cureus.67331

**Published:** 2024-08-20

**Authors:** Najoua Maarad, Mounia Rahmani, Adlaide Taho, Wadii Bnouhanna, Maria Benabdeljlil, Saadia Aïdi

**Affiliations:** 1 Research Team in Neurology, Department of Neurology A and Neuropsychology, Faculty of Medicine and Pharmacy, Specialty Hospital, University Mohammed V, Rabat, MAR

**Keywords:** iron metabolism, ceruloplasmin, anemia, neurodegeneration with brain iron accumulation (nbia), aceruloplasminemia

## Abstract

Aceruloplasminemia (ACP) is a rare genetic disorder that manifests in adulthood due to mutations in the CP (ceruloplasmin) gene, causing iron accumulation and neurodegeneration. Clinically, ACP presents with a range of symptoms, including mild microcytic anemia, diabetes mellitus, liver disease, retinopathy, progressive neurological symptoms such as cerebellar ataxia, involuntary movements, parkinsonism, mood and behavior disorders, and cognitive impairment. We present the case of a 53-year-old female with a history of first-degree consanguinity and a sister with anemia. At six years old, she developed asthenia, leading to multiple hospitalizations for acute hemolytic anemia requiring transfusions and iron therapy. She exhibited later memory disturbances, slowed comprehension, social withdrawal, and school discontinuation. At the age of 51, she developed gait disturbances, unexplained falls, and cognitive decline. One year later, cranial CT revealed a chronic bilateral subdural hematoma. On admission at 53, she had anarthria, right hemiparesis, diffuse rigidity, mouth dystonia, oculomotor paralysis, and intellectual deterioration. MRI showed superficial cortical and leptomeningeal hemosiderin deposits and bilateral signal anomalies in various deep brain regions. EEG revealed paroxysmal anomalies and abdominal MRI indicated hepatic iron overload. Laboratory tests confirmed ACP. This case highlights the rare and severe neurological and systemic manifestations of ACP, emphasizing the importance of early diagnosis and intervention in such degenerative diseases to prevent irreversible neurological complications.

## Introduction

Aceruloplasminemia (ACP) is a rare genetic disorder that manifests in adulthood. It is transmitted in an autosomal recessive pattern and results from mutations in the Ceruloplasmin (CP) gene. This gene, located on 3q24-q25, encodes the CP enzyme, a copper-containing ferroxidase that plays a critical role in cellular iron export and is hypothesized to have a neuroprotective function. In this context, ACP exemplifies a disorder in which the loss of CP function leads to iron accumulation, ultimately resulting in neurodegeneration [1,2]. ACP was initially identified in 1987 in a 52-year-old Japanese woman [3]. The clinical presentation varies and includes conditions such as mild microcytic anemia, diabetes mellitus, retinopathy, liver disease, and progressive neurodegeneration due to iron accumulation in the brain and other organs [2]. Neurological symptoms typically emerge in the fifth decade of life and encompass a broad range of conditions, including cerebellar ataxia, involuntary movements, parkinsonism, mood and behavior disorders, and cognitive impairment [4]. ACP is variably classified among different subgroups of rare diseases, including neurodegeneration with brain iron accumulation (NBIA), atypical microcytic anemias, and non-HFE (hereditary hemochromatosis protein) iron overload syndromes [2].

Diagnosis typically relies on the detection of extremely low or undetectable serum ceruloplasmin levels, along with clinical, biochemical, and radiological evidence of iron overload in target organs, and is confirmed by genetic testing to identify mutations in the CP gene. Many specialties, including internal medicine, hepatology, gastroenterology, endocrinology, neurology, and psychiatry, can serve as entry points for diagnosis. This contributes to the frequently observed diagnostic delay in most cases of ACP. Early treatment is vital to prevent neurological complications, which are frequently irreversible once they develop [2]. Current treatment primarily involves the use of iron-chelating agents, which are generally effective at reducing liver iron accumulation and may help prevent further brain iron deposition. However, they have limited efficacy in patients with existing neurological damage [3,4].

We report here the case of a 53-year-old female patient with ACP, highlighting the challenge and delay in diagnosis and the importance of timely treatment to prevent irreversible neurological damage.

## Case presentation

**Figure 1:**
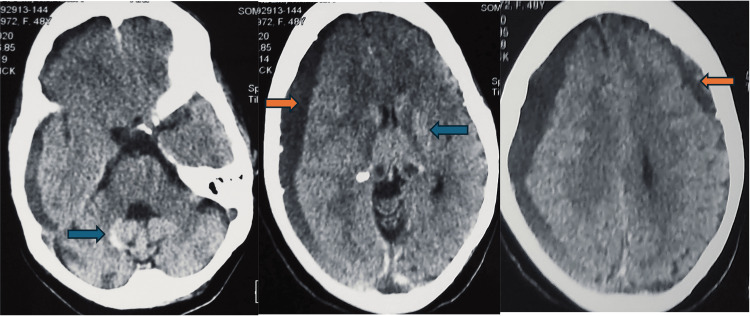
Axial non-contrast brain CT scan showing a right fronto-temporo-parieto-occipital subdural hematoma and a left frontoparietal subdural hematoma (orange arrows) with abnormal high-density areas in the basal ganglia (blue arrows)

A 53-year-old female patient, with a history of first-degree consanguinity and a sister being followed for anemia, initially presented at the age of 6 with cutaneous and mucosal pallor associated with asthenia. She was hospitalized three times in two years for acute hemolytic anemia, requiring multiple transfusions and iron oral therapy. These symptoms were accompanied by memory disturbances, slowed comprehension and movements, and social withdrawal behavior, leading to school discontinuation at the age of 8. Subsequently, the patient exhibited incoherent speech, inappropriate crying, and a progressive inability to perform complex tasks. At the age of 51, she developed gait disturbances with repeated and unexplained falls. Her cognitive impairment worsened too. There were no convulsive seizures or loss of consciousness. Furthermore, one year later, she experienced headaches and right hemiparesis. A cranial CT scan revealed a chronic bilateral subdural hematoma (Figure 1) in the context of anemia at 7.10 g/dl. The patient underwent surgical treatment, specifically a trepanation. The overall improvement post-surgery was limited, likely due to the complexity of the patient's condition. Additionally, the CT scan performed at that time revealed abnormal high-density areas in the basal ganglia. Progression was marked by a gradual worsening of motor function (requiring a person’s assistance for walking and later a wheelchair) and speech difficulties. Upon admission to our service at the age of 53, the patient was anarthric, having right hemiparesis rated at 4/5 with axial and lower limbs rigidity, mouth dystonia during speech, a Babinski sign on the left, a Hoffman sign on the right, complete oculomotor paralysis, and praxis disturbances. She also exhibited intellectual deterioration, repetition difficulties, comprehension issues, especially with complex grammatical instructions, and general health decline.

**Figure 2:**
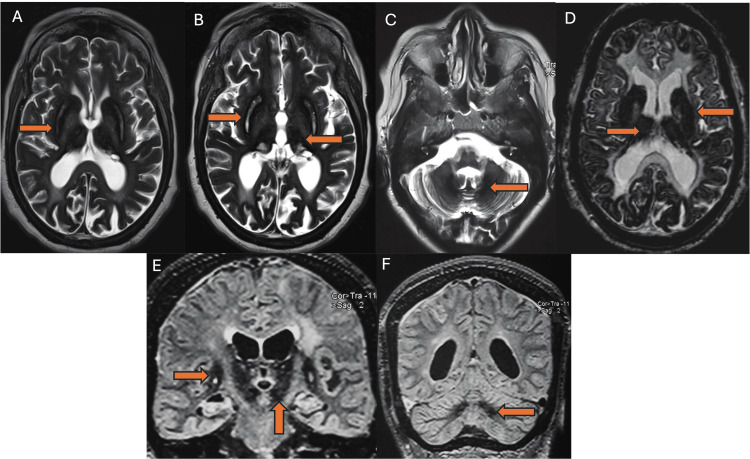
Brain MRI showing bilateral and symmetrical hyposignal in the striatum, globus pallidus, thalami, substantia nigra, and dentate nuclei apparent on axial T2- (A, B, C); SWI- (D) weighted sequences and coronal FLAIR sequences (E, F) SWI: susceptibility-weighted imaging; FLAIR: fluid-attenuated inversion recovery

Cerebral MRI showed bilateral and symmetrical hyposignal in the striatum, globus pallidus, thalami, substantia nigra, red nuclei, and dentate nuclei apparent in all sequences as well as cortical and leptomeningeal hemosiderin deposits in T2, fluid-attenuated inversion recovery (FLAIR) and susceptibility-weighted imaging (SWI) sequences (Figure 2). EEG revealed normal background activity with paroxysmal anomalies in the right fronto-parietal region. Abdominal MRI indicated hepatic iron overload with an estimated concentration of 93.83 µm/g, without suspicious lesions. Biological assessments revealed hypochromic microcytic anemia, with elevated ferritin levels despite low serum iron, as well as very low ceruloplasmin and serum copper levels. The liver and kidney function tests were normal (Table 1). The diagnosis of ACP was based on clinical, biological, and radiological findings. No genetic testing was conducted. Treatment with deferasirox (iron chelator) was proposed but not administered due to financial issues. 

**Table 1:** Patient laboratory results and reference values

Assessment Type	Result	Unit	Reference Range
Hb (Hemoglobin)	9.2	g/dl	12.0-15.5 g/dL
MCV (Mean Corpuscular Volume)	61.2	µm³	79-99 µm³
MCHC (Mean Corpuscular Hemoglobin Concentration)	31	%	32-36%
Ferritin	1537	ng/mL	4-204 ng/mL
Serum Iron	0.27	mg/L	0.4-1.45 mg/L
Ceruloplasmin	0.09	g/L	0.20-0.60 g/L
Serum Copper	1.0	µmol/L	11-20 µmol/L
24-Hour Urinary Copper	23	µg/L	<20 µg/L
ASAT (Aspartate Aminotransferase)	18	UI/L	5-34
ALAT (Alanine Aminotransferase)	23	UI/L	0-55
GGT (Gamma-Glutamyl Transferase)	24	U/L	9-36 U/L
Urea	0.26	g/l	0.15-0.55 g/L
Creatinine	6.8	mg/L	5.7-12.5 mg/L

## Discussion

ACP is a rare autosomal recessive disorder affecting predominantly the Japanese population [5]. Knowledge of ACP epidemiology primarily stems from studies conducted on the Japanese population, where the condition was initially described by Miyajima and colleagues [2,3]. They estimated a prevalence of one in two million among Japanese individuals born from non-consanguineous marriages. This estimation, based on systematic measurements of serum CP levels, does not apply to non-Japanese populations, where the prevalence remains unknown. Other cases have also been documented in European countries, China, and among African American individuals [6,7].

ACP is caused by biallelic mutations in the CP gene, leading to defective CP, a protein crucial for iron homeostasis. CP, consisting of 1,046 amino acids, binds up to six copper atoms and has six structural domains with a three-copper catalytic center essential for its function. Two isoforms of CP exist - a soluble form produced by hepatocytes and a membrane-bound form expressed by various cells, including astrocytes and macrophages. CP collaborates with ferroportin to export iron from cells, converting Fe2+ to Fe3+ for binding to transferrin. The absence of CP impairs this process, causing iron accumulation and oxidative damage, particularly in neurons. Additionally, CP regulates hepcidin, a key hormone in iron metabolism. Disruption of CP leads to neurodegeneration and iron overload in organs like the liver, pancreas, and retina [1]. These findings suggest two mechanisms responsible for cell death depending on the cell type: iron accumulation in astrocytes and functional iron deficiency in neurons. This "iron starvation" in neurons would lead to increased utilization of other forms of iron (non-transferrin-bound iron), causing significant oxidative stress and resulting in neuronal death [8]. Mutations in the CP gene, are often unique and result in absent or reduced ferroxidase activity of CP. To date, 28 missense, 17 frameshift, 13 splicing, and 8 nonsense mutations have been documented. Most cases are associated with homozygous mutations, often linked to a history of consanguinity, although compound heterozygosity has also been reported. Certain mutations, such as Cys338Ser or Ile991Thr, might be linked to residual ferroxidase activity and fewer neurological complications, while Gly631Arg is frequently linked to extrapyramidal symptoms in Caucasian patients [7,9]. There is no clear correlation between genotype and phenotype.

The clinical triad of retinal degeneration, diabetes, and dementia is commonly mentioned, particularly in studies involving Japanese patients with ACP [10]. However, more recently, published case series of non-Japanese populations have highlighted a broader clinical variability, demonstrating that ACP can present with a wider range of symptoms and genetic variations [7,9]. Our reported case of a 53-year-old female with a history of significant early-onset symptoms underscores this heterogeneity. This patient presented with severe anemia and neurological impairments, including cognitive deficits and motor disturbances, which align with the broad spectrum of clinical presentations seen in non-Japanese populations. The early onset of anemia and subsequent neurological decline is indicative of the disease's progression and demonstrates the critical role of early diagnosis and management.

Based on several descriptions, the clinical presentation of ACP that typically leads neurologists to diagnose this condition includes cerebellar signs such as dysarthria, trunk and limb ataxia, and involuntary movements like dystonia, chorea, and tremors, usually appearing between the ages of 40 and 60 [2]. Respectively, dystonia is observed in more than 40% and chorea/choreoathetosis in approximately 25% of cases [3]. Cognitive-psychiatric changes and extrapyramidal signs appear more frequently and earlier in Caucasians than in Japanese patients. However, cognitive alterations, such as apathy, memory loss, and behavioral difficulties have low specificity and are often underestimated. These differences suggest that genetic modifiers, expressed differently in Caucasian and Japanese populations, influence the disease's phenotypic expression [2,9]. In our case, the patient displayed progressive significant motor and speech impairments. This progression is consistent with the typical neurological manifestations of ACP, further complicated by the patient's severe cognitive decline and social withdrawal, highlighting the need for comprehensive neurological assessments in suspected cases.

Additionally, many studies highlight the presence of moderate anemia as an initial biological sign of ACP. This anemia, often traceable to childhood, rarely leads to early presymptomatic diagnosis [7,11]. Our patient's history of severe microcytic anemia requiring multiple transfusions and iron therapy from a young age, coupled with her neurological symptoms, illustrates the complex interplay between hematological and neurological manifestations in ACP. The paradoxical nature of anemia, often microcytic with low transferrin saturation and hyperferritinemia, is usually present in several cases.

Patients with ACP also commonly present with diabetes or abnormal glucose tolerance. Autopsy studies have revealed significant iron accumulation within the endocrine portion of the pancreas, with a marked reduction in the beta-cell population within the islets of Langerhans [12]. Retinopathy, although not found in our patient, is frequently reported in 70% of Japanese patients though it rarely causes significant visual impairment. In Western populations, its prevalence is much lower. It is characterized by epithelial depigmentation, alternating atrophic and hypertrophic areas, and small yellowish lesions (nodular drusen) mainly in the posterior pole of the retina. Retinal pigment, epithelial cells, and the neural retina are often loaded with iron [13]. The absence of retinopathy in our case aligns with the lower prevalence observed in non-Japanese series.

In an international study evaluating 11 new ACP families with 13 affected individuals, neurological symptoms were predominant, appearing in 75% of the patients. These symptoms ranged from severe neurological and psychiatric syndromes to milder cognitive dysfunctions. The study also noted that diabetes mellitus and retinopathy were observed in 46.2% and 40% of cases, respectively. Anemia was common, found in 84.6% of patients, with 61.5% exhibiting microcytosis. High serum ferritin levels and very low or undetectable ceruloplasmin levels were consistent findings. This extensive neurological involvement underscores the critical impact of ACP on the central nervous system, highlighting the need for early diagnosis and intervention to manage the neurodegenerative aspects of the disease [1]. Even though it is a rare condition, the presence of atypical diabetes in a young patient, along with biological anomalies (microcytic anemia) and psychiatric disorders, should prompt consideration of ACP.

Other laboratory investigations in ACP reveal low or absent serum CP, elevated serum ferritin, low serum iron and copper, and normal urinary copper levels [12]. When ACP is suspected based on neurological symptoms, performing a brain MRI with specific sequences is crucial, as neuroimaging studies reveal distinct findings. T1 and T2-weighted images typically reveal low intensities indicative of iron accumulation in the liver and various brain regions, including the basal ganglia, thalamus, and dentate nucleus. T2* weighted brain MRI shows marked hypointensity in the basal ganglia (caudate nucleus, putamen, and pallidum) and the thalamus, which are hallmark features of ACP. These imaging patterns underscore the extensive neurodegeneration and iron deposition and help differentiate ACP from other NBIA disorders like PKAN, neuroferritinopathy, and infantile neuroaxonal dystrophy. Additionally, T2* weighted images reveal diffuse hypointensities in the cerebral and cerebellar cortices, indicating more widespread iron accumulation than previously reported [2,14]. Liver biopsy results show important iron accumulation, with levels exceeding 1000 mg/g dry weight, within both hepatocytes and reticuloendothelial cells. Despite this significant iron overload, hepatic architecture and histology remain normal, without signs of cirrhosis or fibrosis. Additionally, copper accumulation levels are normal [12]. Our case exemplifies these typical findings. The patient's laboratory results showed the characteristic low serum ceruloplasmin, elevated ferritin, decreased serum iron, and low serum copper. Brain MRI findings were consistent with ACP, showing hypointensities in the basal ganglia, thalamus, and cerebellar cortex. Such findings are typical imaging patterns of ACP, hence confirming the diagnosis.

Current knowledge on the treatment of ACP is largely based on case reports, with the common approach in most cases being iron-chelating agents. Deferiprone is particularly noteworthy due to its ability to cross the blood-brain barrier, unlike deferoxamine and deferasirox. Although these drugs have demonstrated potential in lowering serum ferritin and liver iron levels, their effectiveness in reducing brain iron accumulation and alleviating neurological symptoms remains a subject of ongoing debate. Early treatment may slow neurodegeneration, but effectiveness is hard to assess due to inconsistent clinical and MRI descriptions. Poor tolerance and worsening anemia limit the long-term use of chelators. Phlebotomies are less effective due to impaired iron mobilization. Combining chelation with fresh frozen plasma has shown transient benefits. Antioxidants like vitamin E and zinc sulfate, as well as tetracyclines like minocycline, offer potential benefits. Experimental treatments in mice with human CP administration have shown promise, suggesting possible future human applications [11,12].

Overall, our case highlights the critical importance of the early and accurate diagnosis of ACP, especially in patients with early-onset anemia and neurological symptoms. It underscores the diverse clinical manifestations of ACP and the need for a multidisciplinary approach to diagnosis and treatment.

## Conclusions

ACP is a rare autosomal recessive disorder with significant clinical heterogeneity. It is characterized by iron accumulation in multiple organs, leading to diverse neurological and systemic symptoms. Our case underscores the importance of early and accurate diagnosis, particularly in patients with early-onset anemia. Despite promising results with iron-chelating agents like deferiprone, the efficacy of these treatments on brain iron accumulation and neurological manifestations remains uncertain, emphasizing the need for further research.

A multidisciplinary approach, including regular monitoring and a combination of therapeutic strategies, is crucial for managing the complex interactions between hematological and neurological manifestations in ACP. Advances in understanding the pathophysiology and development of targeted treatment will be crucial for improving the outcomes of patients with this challenging condition. 

## References

[1] Vila Cuenca M, Marchi G, Barqué A (2020). Genetic and clinical heterogeneity in thirteen new cases with aceruloplasminemia. Atypical anemia as a clue for an early diagnosis. Int J Mol Sci.

[2] Marchi G, Busti F, Lira Zidanes A, Castagna A, Girelli D (2019). Aceruloplasminemia: a severe neurodegenerative disorder deserving an early diagnosis. Front Neurosci.

[3] Miyajima H (2015). Aceruloplasminemia. Neuropathology.

[4] Piperno A, Alessio M (2018). Aceruloplasminemia: waiting for an efficient therapy. Front Neurosci.

[5] Di Meo I, Tiranti V (2018). Classification and molecular pathogenesis of NBIA syndromes. Eur J Paediatr Neurol.

[6] Miyajima H, Kohno S, Takahashi Y, Yonekawa O, Kanno T (1999). Estimation of the gene frequency of aceruloplasminemia in Japan. Neurology.

[7] Pelucchi S, Mariani R, Ravasi G (2018). Phenotypic heterogeneity in seven Italian cases of aceruloplasminemia. Parkinsonism Relat Disord.

[8] Bishop GM, Dang TN, Dringen R, Robinson SR (2011). Accumulation of non-transferrin-bound iron by neurons, astrocytes, and microglia. Neurotox Res.

[9] Vroegindeweij LH, Langendonk JG, Langeveld M (2017). New insights in the neurological phenotype of aceruloplasminemia in Caucasian patients. Parkinsonism Relat Disord.

[10] Miyajima H, Takahashi Y, Kono S (2003). Aceruloplasminemia, an inherited disorder of iron metabolism. Biometals.

[11] Zanardi A, Conti A, Cremonesi M (2018). Ceruloplasmin replacement therapy ameliorates neurological symptoms in a preclinical model of aceruloplasminemia. EMBO Mol Med.

[12] Kono S (2013). Aceruloplasminemia: an update. Int Rev Neurobiol.

[13] Wolkow N, Song Y, Wu TD, Qian J, Guerquin-Kern JL, Dunaief JL (2011). Aceruloplasminemia retinal histopathology and iron-mediated melanosome degradation. Arch Ophthalmol.

[14] Grisoli M, Piperno A, Chiapparini L, Mariani R, Savoiardo M (2005). MR imaging of cerebral cortical involvement in aceruloplasminemia. AJNR Am J Neuroradiol.

